# The complete chloroplast genome of *Artemisia hallaisanensis* Nakai (Asteraceae), an endemic medicinal herb in Korea

**DOI:** 10.1080/23802359.2018.1450680

**Published:** 2018-03-14

**Authors:** Chae Eun Lim, Goon-Bo Kim, Se-A Ryu, Hee-Ju Yu, Jeong-Hwan Mun

**Affiliations:** a National Institute of Biological Resources, Incheon, Korea;; b Department of Life Science and Bioinformatics, Myongji University, Yongin, Korea;; c Department of Life Science, The Catholic University of Korea, Bucheon, Korea

**Keywords:** *Artemisia hallaisanensis*, endemic, chloroplast genome, variation

## Abstract

We determined the complete chloroplast DNA sequence of *Artemisia hallaisanensis* Nakai, an endemic herbal species distributed on Jeju Island, Korea. The chloroplast DNA is 151,015 bp in length and encodes 4 rRNA, 30 tRNA, and 80 protein-coding genes. Phylogenetic analysis and sequence comparison of protein-coding genes with other *Artemisa* chloroplast DNAs revealed that the chloroplast genome of *A. hallaisanensis* is closely related to that of *A. capillaris.* Additionally, a unique 9 bp deletion in *ycf1* gene is specific to *A. hallaisanensis*.

*Artemisia* is the largest genus in the tribe Anthemideae of the Asteraceae family consisting of more than 500 species that are widely distributed in temperate regions of the northern hemisphere (Oberprieler et al. 2009; Vallès et al. 2011). The *Artemisia* species have been well known as medicinal herbs in Asia for their high accumulation of essential oils and terpenoids (Wagner 1977). However, taxonomic delimitation of several taxa based on morphological characteristics has been controversial. Molecular marker techniques could solve this issue by providing better criteria for classification of *Artemisia* species (National Institute of Biological Resources 2015).

In this study, we determined the complete chloroplast (cp) genome of *A*. *hallaisanensis*, an endemic species of Korea showing a very limited distribution on Jeju Island (National Institute of Biological Resources 2013). The specimen was collected from Jeju Island (33°21′34″N, 126°30′37.9″E) and deposited in the National Institute of Biological Resources with an accession number NIBR-VP0000538771. A total of 26 million paired-end reads were generated using the Illumina MiSeq platform. A circular DNA was assembled using NOVOPlasty program (version 2.5.9; Dierckxsens et al. 2017) with *rbcL* and *rpoC2* genes of *A. annua* cp DNA (Shen et al. 2017) as seed sequences. The assembled cp DNA (Genbank accession MG951490) was 151,015 bp in length with 37.5% overall GC content. The genome structure was highly similar to the reported *Artemisia* cp DNAs, consisting of two IRs (24,951 bp), one LSC (82,823 bp), and one SSC (18,290 bp). Genome annotation using Geneious program (version 11; Kearse et al. 2012) predicted a total of 114 genes (4 rRNA, 30 tRNA, and 80 protein-coding genes), of which 18 genes (4 rRNA, 7 tRNA, and 7 protein-coding genes) were duplicated in two IRs. Twenty genes (6 tRNA and 14 protein-coding genes) contained one or two introns. The maximum likelihood tree constructed using 77 non-redundant protein-coding genes in cp DNAs from 10 *Artemisia* species and two outgroup species (*Aster spathulifolius* and *Helianthus annuus*) indicated that the cp genome of *A. hallaisanensis* is closely related to that of *A. capillaris* in section Dracunculus (Figure 1). Moreover, sequence comparison identified distinct sequence variations in *ycf1* gene of *A. hallaisanensis* and *A. capillaris*. Two deletions (21 bp and 6 bp, respectively) in *ycf1* were evident in both taxa. Interestingly, *A. hallaisanensis* contains additional 9 bp deletion in *ycf1*. This unique sequence divergence identified in the cp DNA will serve as a molecular basis for identification of this species as well as phylogenetic study of *Artemisia*.

**Figure 1.**
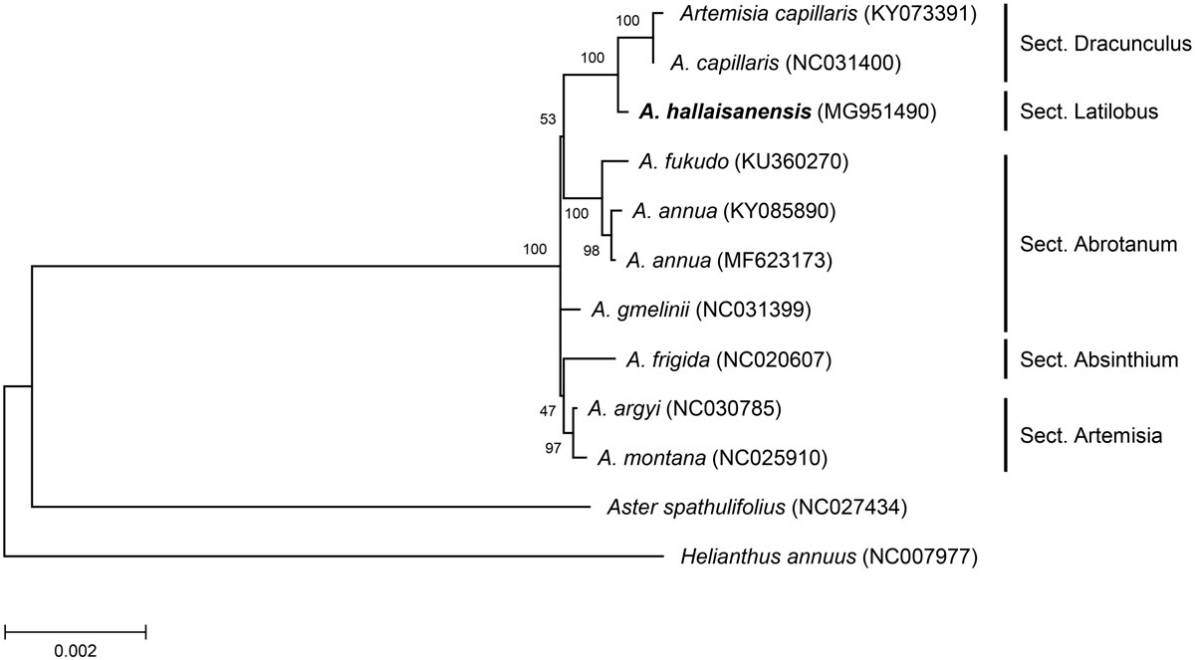
Maximum likelihood tree based on the chloroplast protein-coding genes of 12 taxa including *A. hallaisanensi*s. Nucleotide sequences of 77 non-redundant protein-coding genes from publically available *Artemisia* species as well as two distantly related taxa (*Aster spathulifolius* and *Helianthus annuus*) from Asteraceae were aligned and used for maximum likelihood phylogenetic analysis in MEGA7 (Kumar et al. 2016) with bootstrap test of 1000 replications. The NCBI accession numbers of chloroplast DNA sequences used in this study are presented in parentheses. The scale bar represents the number of substitutions per site.
